# Kidney Transplantation in the Diabetic Patient

**DOI:** 10.3390/jcm4061269

**Published:** 2015-06-09

**Authors:** María José Pérez-Sáez, Julio Pascual

**Affiliations:** Department of Nephrology, Hospital del Mar, Barcelona 08003, Spain; E-Mail: mjoseperezsaez@gmail.com

**Keywords:** kidney transplantation, diabetes, survival

## Abstract

Diabetes mellitus is one of the most important causes of chronic kidney disease (CKD). In patients with advanced diabetic kidney disease, kidney transplantation (KT) with or without a pancreas transplant is the treatment of choice. We aimed to review current data regarding kidney and pancreas transplant options in patients with both type 1 and 2 diabetes and the outcomes of different treatment modalities. In general, pancreas transplantation is associated with long-term survival advantages despite an increased short-term morbidity and mortality risk. This applies to simultaneous pancreas kidney transplantation or pancreas after KT compared to KT alone (either living donor or deceased). Other factors as living donor availability, comorbidities, and expected waiting time have to be considered whens electing one transplant modality, rather than a clear benefit in survival of one strategy *vs.* others. In selected type 2 diabetic patients, data support cautious utilization of simultaneous pancreas kidney transplantation when a living kidney donor is not an option. Pancreas and kidney transplantation seems to be the treatment of choice for most type 1 diabetic and selected type 2 diabetic patients.

## 1. Introduction

In the last few decades, the developed world has experienced a huge impact of chronic kidney disease (CKD) in the diabetic patient. According to a 2014 report (with 2012 data) of the *US Renal Data System* (USRDS), 44% of total end-stage renal disease (ESRD) causes are diabetic nephropathy [1]. The absolute number is about 50,000 patients in this country. However, the incidence of ESRD in diabetics differs between countries: the percentage ranges between 16% in the Netherlands, 23% in the United Kingdom, and 66% in Singapore [1]. The incidence of diabetics with ESRD in Spain is about 21%, as of 2013 [2]. Despite the alarming data, some progress has been achieved in order to reduce the impact of this entity, in line with the objectives set by the initiative of CKD within the program *Healthy People 2020* [3]. The USRDS registry confirms that, while in the 1990s the incidence increased in an exponential way, it remained stable in the following decade [1]. These rates have been stable in recent years among younger individuals, but they have declined in older individuals in most racial groups. Among diabetic patients, an overall reduction in those developing ESRD has also been observed: the rate of new ESRD cases with diabetes listed as the primary cause has declined since 2006 [1].

In addition to the USRDS registry, an important database including 25,000 diabetic patients with ESRD in US was described a few years ago [4]. More than 95% of all diabetics were type 2, while type 1 diabetics represented only 5%, a similar distribution to that described in the general diabetic population. Type 1 diabetics were younger, more frequently Caucasian, with more time on dialysis, more time with a native fistula, and with poor phosphorus and glycemic control [4].

## 2. Transplantation Therapy Options

Kidney transplantation is associated with better survival outcome compared to dialysis. Wolfe *et al.* analyzed US registry in terms of mortality of dialysis patients on the waiting list compared to deceased donor KT patients [5]. KT was associated with lower mortality rate in 18 months, with an evident benefit from the 244th day after transplant. The projected increase in life expectancy due to KT was 10 years, particularly in diabetic population that presented a poor prognosis remaining on dialysis. These findings have been confirmed in other registries [6,7,8]. One-year KT survival in diabetic patients is now approaching 88% for deceased donors (DD) and 96% for living donors (LD) [9]. Pancreas transplantation has become increasingly successful in recent years due to advances in surgical outcomes and immunosuppression [10,11]. One-year pancreas graft survival is now nearly 95% when performed as a simultaneous pancreas-kidney transplant (SPK), and 86% when performed as a pancreas after KT (PAK) [12]. In one single center, mortality risk for diabetic patients was 2–3-fold lower in those who received a pancreas transplant [13]. 

## 3. Results of Transplantation in Type 1 Diabetic Patients

Type 1 diabetic patients with an estimated glomerular filtration rate (eGFR) below 30 mL/min must start an information and decision process in order to manage their advanced CKD with one of the available modalities. This process accelerates when eGFR decreases below 20 mL/min. With the evaluation of only type 1 diabetic patients in a complex decision model published by Markov in 2003, donor living KT was the best therapeutic technique for these patients, with 18 gained life-years. These results were better than PAK transplantation, SPK transplantation, DDKT, and dialysis (Table 1) [14]. It is undisputed that KT is better than dialysis in terms of quality of life and survival and dialysis is the alternative only when KT is contraindicated.

**Table 1:** Gained life-years and gained quality life-years obtained with different therapeutic choices in type 1 diabetic patient, according to Markov model [14].

	Life-Years	Quality Life-Years
Living donor KT	18.3	10.3
PAK transplantation	17.2	10
SPK transplantation	15.7	9.1
Deceased donor KT	11.4	6.5
Dialysis	7.8	4.5

KT, kidney transplant; PAK, pancreas after kidney; SPK, simultaneous pancreas-kidney.

However, there is not randomized controlled data for any form of transplantation in patients with diabetes and CKD stage 5. DDKT is the modality that achieves worse outcomes. Therefore, it seems reasonable that living donor KT to be the first option if the patient is a KT candidate and has a potential donor. The longer the time waiting for a transplant on dialysis, the larger the advantage of LDKT over deceased SPK. This superiority consolidates and increases decisively when, in addition, the candidate is expected to receive PAK transplantation. During 2000–2007, almost 12,000 type 1 diabetic patients on the waiting list for a KT and pancreas transplant underwent a first transplant: 47% SPK, 29% LDKT, and 24% DDKT [15]. From 3461 patients that received a LDKT, 807 (23%) subsequently received a pancreas, while 77% of them did not. Even adjusting by confounding factors, five-year patient and kidney graft survival of PAK were significantly better than in SPKT. On the other hand, pancreas graft survival was better in SPK than in PAK, even after censoring for death with function [15]. No differences were detected in patient survival between SPK and PAK since the moment that PAK patients received their pancreas. Previous studies had described better graft survival in LDKT than SPK, with similar patient survival [16]. A recent study compared 0-HLA mismatched DDKT recipients to SPK and LDKT recipients. Comparable graft and patient survival was observed, implying that if waiting time for an SPK was expected to be long and/or put the candidate at excessive risk while awaiting the transplant, the opportunity of a 0 mismatched DDKT is a reasonable approach [17].

However, some studies follow-up more than ten years have observed better patient survival with SPK than PAK or LDKT, mainly due to the cardiovascular mortality reduction experienced with a functioning pancreas implanted since the moment of KT [18,19,20]. Although five-year results seemed to give advantage to LDKT alone over deceased SPK [16,18], survival is similar when 10-year outcomes are analyzed. When the follow-up is even longer (until 18–20 years), the functioning pancreas since the very beginning seems to be essential to make the difference, and the best survival is with SPK [5,16,18,19,20,21,22,23,24,25,26] (Table 2). More than 10 years data is only available from the *Collaborative Transplant Study* registry [18,20] and inclusion bias or the possibility that SPK patients were healthier than LDKT cannot be discarded. 

In addition, despite that some studies have highlighted the importance of early pancreas allograft function in longer-term outcomes, the risks associated with early pancreatic loss have been shown in a large retrospective analysis to be associated with worse kidney allograft and patient survival. Those with pancreas graft loss within 90 days had a 70% higher risk of kidney graft failure and greater than two-fold increased risk of death at three years compared to those with functioning pancreas allograft at 90 days [27].

**Table 2:** The impact of glycemic control achieved with a pancreas graft on renal graft and patient survival.

Observation Period	Patient Survival	Kidney Graft Survival	Source/Database	Ref
Until 244th day	Dialysis > DDKT	Not applicable	USRDS	[5]
3–6 years	SPK = DDKT	SPK = DDKT	UNOS	[20]
0–7 years	LDKT > SPK = DDKT	LDKT > SPK = DDKT	OPTN/UNOS	[15]
Until 7 years	SPK (P+) > LDKT > DDKTLDKT > SPK	SPK (P+) > LDKT > DDKTLDKT > SPK	SRTR	[18]
	(P-) = DDKT	(P-) = DDKT		
2–9 years	SPK = DDKT	SPK = DDKT	UNOS	[21]
0–10 years	SPK > DDKT	SPK = DDKT	Irish Center	[22]
Until 10 years	LDKT = SPK > DDKT	LDKT = SPK > DDKT	US Center	[23]
Mean = 4.8 years	LDKT = SPK > DDKT	LDKT = SPK > DDKT	UNOS	[24]
0–12 years	Not applicable	SPK > DDKT	OPTN/UNOS/SRTR	[25]
18 years	SPK > LDKT > DDKT	SPK = LDKT > DDKT	CTS	[18]
20 years	SPK > LDKT > DDKT	SPK > LDKT > DDKT	CTS	[20]

Adapted from Morath *et al.* [20]; P+, 1 year functioning pancreas; P-, 1 year non-functioning pancreas; LDKT, living donor kidney transplant; DDKT, deceased donor kidney transplant; SPK, simultaneous pancreas-kidney.

There is a high risk for selection bias in the observational data, as the access to the waiting list is hampered for patients with diabetes. This is consistent with the observation that most guidelines recommend more intense screening, especially for cardiovascular disease, in patients with diabetes [28]. As a result, the outcome results observed after transplantation in diabetics are only valid for those without substantial comorbidity, *i.e.*, who have passed pre-transplant screening procedures [28]. This might be at play in the observed results of SPK transplantation for patients with type 1 diabetes. SPK is mostly performed at high-volume centers, and this most likely affects generalizability of outcomes by referral bias. The healthiest patients are also likely to be allocated to SPK, receive the highest quality organs [29] and get more often a pre-emptive transplant [30].

**Figure 1:**
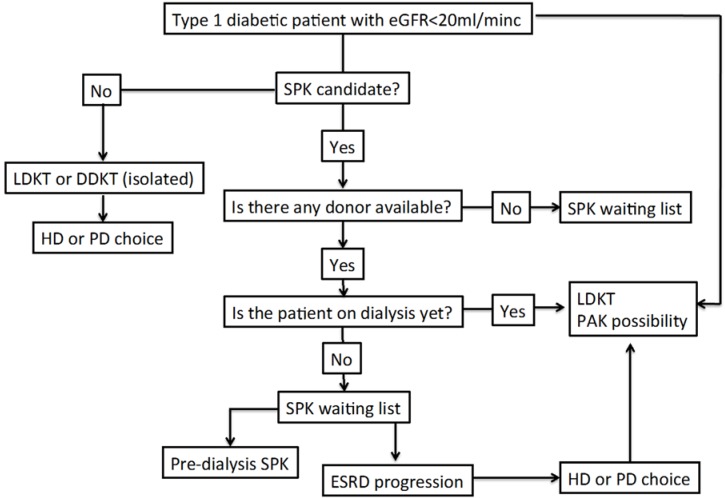
Diabetic patient treatment management proposed algorithm. eGFR, estimated glomerular filtration rate; LDKT, living donor kidney transplant; DDKT, deceased donor kidney transplant; SPK, simultaneous pancreas-kidney; PAK, pancreas after kidney; HD, hemodialysis; PD, peritoneal dialysis; ESRD, end-stage renal disease.

In summary, LDKT with or without PAK and SPK are better alternatives than DDKT. When a living donor exists, LDKT is an excellent choice as good as SPK if the option of PAK is possible in a short period of time. There are some authors that recommend isolated LDKT and then an evaluation for PAK in patients with a living donor as a preferred option to SPK waiting list entrance [31]. A detailed treatment algorithm is proposed in Figure 1.

Pancreas transplant has allowed reverting many complications, such as neuropathy [32] and nephropathy [33], in type 1 diabetic patients. Pancreatic islet transplant is considered apart from this conclusion, as it is an experimental procedure for much selected cases and its effects on complications improvement have not been studied in a rigorous way. Most of them are performed in diabetic patients without nephropathy and sporadically in KT patients who are candidates to a pancreatic graft transplant thereafter. The main problem is the adequate isolation and preservation of the islets and the enough infusion into the recipient to allow insulin-independency for long periods of time [34].

## 4. Results of Transplantation in Type 2Diabetic Patients

Type 2 diabetic patients without evident contraindication for KT may be considered for LDKT if a suitable donor is available. If not, they should be offered to be included on a KT waiting list for DDKT. After KT, these patients have a superior life expectancy than on dialysis [5], and a similar graft survival than non-diabetic patients (adjusting by age and censoring by death with functioning graft) [35,36]. However, five-year patient survival rate is lower than in non-diabetics (70% *vs.* 93%), due to older age and a relevant excess of cardiovascular and infectious mortality [37]. 

SPK indication in this population is controversial. The only study available that analyzes a significant number of type 2 diabetic patients concludes that SPK could be a very positive option for them [38]. There were a total of 24,084 candidates included on SPK waiting list in US from 1992–2007. Among them, 7.8% were type 2 diabetics, with an increase from 3% in 1994 to 8% (*n* = 149) in 2000, and further stabilization in 8%–10%. From 6756 SPK performed between 2000 and 2007, 8.6% were in type 2 diabetes patients, with an important variability among US regions [38]. Although type 2 diabetic patients showed worse survival than type 1 diabetics, the multivariate analysis showed that type 2 diabetes was not a significant risk factor for mortality in SPK. Risk factors for mortality in type 2 diabetic patients who underwent a SPK were age and dialysis time. Interestingly, five-year pancreas survival was similar between both diabetes groups, although it is necessary to confirm these data in long-term studies. While this demonstrated that pancreas transplant was feasible and successful in selected type 2 diabetic patients, this did not clarify whether SPK was a superior alternative to DDKT or LDKT options. 

Recently, two studies support the option of SPK for type 2 diabetic patients. Long-term data suggest similar patient and graft survival regardless of diabetes type after risk stratification [39]. In another single-center review of 216 SPK transplants from Europe, 21 of them had a diagnosis of type 2 diabetes [40]. Type 1 patients differed from type 2 patients: whereas three-quarters of type 1 patients did not have findings of vascular disease, three quarters of patients with type 2 did. The most common cause of pancreatic graft loss was rejection in type 1 patients *vs.* patient death in type 2 patients. Importantly, patient and pancreas graft survival were not different at five years. Overall patient survival in the type 2 SPK patients was superior to those patients undergoing KT alone [40]. Taken together, there is clearly a group of type 2 diabetic patients who benefit from SPK, and both short-term and long-term outcomes are commensurate with type 1 patients. 

When comparing type 2 recipients aged 18–59 years old with a BMI of 18–30 kg/m^2^, five-year patient survival was highest after LDKT (87.3%) followed by SPK (82.0%) and DDKT (75.5%) [41]. This suggests that LDKT may be the superior transplant at least in the medium-term, with the caveat of the potential longer-term benefits from SPK that may accrue over time.

In November 2011, the United Network for Organ Sharing (UNOS) approved eligibility criteria for pancreas transplant candidacy. Eligible SPK candidates are defined as diabetic patients on insulin treatment with a C-peptide ≤ 2 ng/mL, or C-peptide ≥ 2 ng/mL and BMI < 28 kg/m^2^, to permit and define eligibility for type 2 diabetes candidates. This BMI cut-off may be adjusted based upon the percentage of candidates in this category every six months. These patients will be given allocation priority over other DDKT candidates when a kidney/pancreas donor is identified, thus gaining benefit from a shorter waiting time. This policy may impact access to pancreas transplant for type 1 recipients. Ongoing assessments of the degree to which type 2 patients access the pancreas waiting list will require ongoing monitoring, particularly considering the lack of data demonstrating a clear survival advantage of SPK over DDKT.

## 5. Predialysis Kidney Transplant Results

Beyond the question if SPK is better than LDKT with a PAK, KT previous to dialysis is superior in terms of patient survival than KT with the patient on dialysis. This difference increase is more and more accentuated as the patients stay on dialysis before undergoing a KT [42,43,44]. Mange *et al.* showed that LDKT without previous dialysis was associated to 52% lower risk of graft loss during the first year after transplant than patients receiving the LDKT while on dialysis. The benefit was even higher in later years [36]. Curiously, the risk of acute rejection was higher in patients that had been on dialysis than in predialysis patients. After this study, Kasiske *et al.* showed similar results, and not only in LDKT receptors but also in DDKT patients, with a benefit in kidney and patient survival [45]. This benefit is evident with both genetically related and unrelated LDKT [45]. More recently, the advantage of receiving a predialysis LDKT or SPK has been confirmed, without any difference when the predialysis KT is from a deceased donor [46].

The advantages of predialysis SPK over postdialysis SPK are significant in both US and European studies [47], with a better patient and graft survival. However, this difference is only evident from the first year (kidney) and 10 years after transplant (patient). A US study compared the four modalities of predialysis KT in type 1 diabetic patients: predialysis SPK (*n* = 1402), pancreas transplant after predialysis LDKT (*n* = 389), predialysis LDKT without pancreas (*n* = 289) and predialysis DDKT (*n* = 112) [48]. After six years, patient survival was excellent (91.1%, 89.4%, 84.9% and 81.2%, respectively), significantly higher with SPK or pancreas transplant after LDKT than with predialysis DDKT. A registry study of 2776 PAK recipients demonstrated excellent kidney graft survival (69% at 10 years) in those with a GFR < 40 mL/min before PAK [49].

Therefore, the benefit of predialysis LDKT in type 1 diabetic patients is remarkable. Efficient strategies to perform early KT are needed. The achievement of a LDKT is of particular importance; especially in situations where waiting time for a DDKT is long. Similarly, the accumulation of dialysis time while awaiting either an SPK or LDKT is associated with diminished post-transplant survival with either modality [50]. However, the time to do it should not be advanced in an unjustified way, as it has been shown that kidney survival did not improve undergoing transplant with eGFR > 15 mL/min compared to right before dialysis initiation (eGFR < 15 mL/min) [51].

## 6. Summary and Conclusion: The Way to the Best Quality in Diabetic Patient Care with CKD

The prognosis of the diabetic patient with ESRD and without the possibility of LDKT or early DDKT is still poor. It is necessary to face a list of problems with prognostic implications and whose proper management may improve life expectancy and life quality.

Cardiovascular disease is the most frequent cause of morbidity and mortality in this population. Peripheral vascular disease, infections and malnutrition strongly impact in prognosis as well. Therefore, the adequate control of blood pressure [44], glycemia [45], lipid profile [52,53] and nutrition [54] are essential. 

Given the narrow but consistent advantages of an added pancreas transplant, either as an SPK or PAK, over KT alone, the decision-making for a specific individual regarding transplant options will most likely focus upon the individual’s morbidity and perceived quality of life. The ongoing diabetes care, the difference in waiting time for an SPK rather than for a KT (typically via a living donor), and the characteristics and allocation policies that govern the transplant center are crucial factors. Given the mortality data and pancreas graft survival data provided above, it is reasonable to pursue SPK *versus* LDKT if it can be done pre-emptively. However, if the patient is nearing or entering dialysis, LDKT should be reconsidered given the associated morbidity and potential mortality risk that is assumed while waiting for transplant (Figure 1). Pancreas transplantation, either as an SPK or PAK, may improve patient survival, reduce the risk of kidney graft failure, and improve quality of life in patients with type 1 diabetes mellitus and kidney disease. Preliminary evidence shows that technical and early outcomes are similar in selected patients with type 2 diabetes mellitus. Balancing the benefits of the added pancreas transplant *versus* the risks of waiting should be assessed on an individual/center basis. In general, the balance will favor an SPK transplant when patients are not on dialysis, and LDKT/PAK if patients are receiving dialysis.

## References

[1] United States Renal Data System The 2014 Annual Date Report. Chapter 1: Incidence, prevalence, patient characteristics, and treatment modalities. http://www.usrds.org/2014/view/.

[2] Sociedad Española de Nefrología (S.E.N.) Informe de 2013 (Congreso de Barcelona, 2014). http://www.senefro.org/modules.php?name=webstructure&amp;idwebstructure=128.

[3] Healthypeople.gov. 2020 Topics & Objectives. Chronic Kidney Disease. http://www.healthypeople.gov/2020/.

[4] Williams M.E., Lacson E., Teng M., Ofsthum N., Lazarus J.M. (2006). Hemodialyzed type I and type II diabetic patients in the US: Characteristics, glycemic control and survival. Kidney Int..

[5] Wolfe R.A., Ashby V.B., Milford E.L., Ojo A.O., Ettenger R.E., Agodoa L.Y., Held P.J., Port F.K. (1999). Comparison of mortality in all patients on dialysis, patients on dialysis awaiting transplantation and recipients of first cadaveric transplant. N. Engl. J. Med..

[6] Schnuelle P., Lorenz D., Trede M., van der Woude F.J. (1998). Impact of renal cadaveric transplantation on survival in end-stage renal failure: Evidence for reduced mortality risk compared with hemodialysis during long-term follow-up. J. Am. Soc. Nephrol..

[7] McDonald S.P., Russ G.R. (2002). Survival of recipients of cadaveric kidney transplants compared with those receiving dialysis treatment in Australia and New Zealand 1991–2001. Nephrol. Dial. Transplant..

[8] Lloveras J., Arcos E., Comas J., Crespo M., Pascual J. (2015). A paired survival analysis comparing hemodialysis and kidney transplantation from deceased elderly donors older than 65 years. Transplantation.

[9] Matas A.J., Smith J.M., Skeans M.A., Lamb K.E., Gustafson S.K., Samana C.J., Stewart D.E., Snyder J.J., Israni A.K., Kasiske B.L. (2013). OPTN/SRTR 2011 Annual Data Report: Kidney. Am. J. Transplant..

[10] Fridell J.A., Powelson J.A., Sanders C.E., Ciancio G., Burke G.W., Stratta R.J. (2011). Preparation of the pancreas allograft for transplantation. Clin. Transplant..

[11] Heilman R.L., Mazur M.J., Reddy K.S. (2010). Immunosuppression in simultaneous pancreas-kidney transplantation: Progress to date. Drugs.

[12] Kandaswamy R., Skeans M.A., Gustafson S.K., Carrico R.J., Tyler K.H., Israni1 A.K., Snyder J.J., Kasiske B.L. (2015). OPTN/SRTR 2013 Annual Data Report: Pancreas. Am. J. Transplant..

[13] VanDellen D., Worthington J., Mitu-Pretorian O.M., Ghazanfar A., Forgacs B., Pararajasingam R., Campbell B., Parrott N.R., Augustine T., Tavakoli A. (2013). Mortality in diabetes: Pancreas transplantation is associated with significant survival benefit. Nephrol. Dial. Transplant..

[14] Knoll G., Nichol G. (2003). Dialysis, kidney transplantation or pancreas transplantation for patients with diabetes mellitus and renal failure: A decision analysis of treatment options. J. Am. Soc. Nephrol..

[15] Poommipanit N., Sampaio M.S., Cho Y., Young B., Shah T., Pham P.T., Wilkinson A., Danovitch G., Bunnapradist S. (2010). Pancreas after living donor kidney *versus* simultaneous pancreas-kidney transplant: An analysis of the organ procurement transplant network/United Network of Organ Sharing database. Transplantation.

[16] Young B.Y., Gill J., Huang E., Takemoto S.K., Anastasi B., Shah T., Bunnapradist S. (2009). Living donor kidney *versus* simultaneous pancreas-kidney transplant in type I diabetics: An analysis of the OPTN/UNOS database. Clin. J. Am. Soc. Nephrol..

[17] Kamgar M., Huang E., Kamgar M., Nata N., Leeaphorn N., Kalantar-Zadeh K., Bunnapradist S. (2012). Zero-mismatch deceased-donor kidney *versus* simultaneous pancreas-kidney transplantation. Transplantation.

[18] Morath C., Zeier M., Dohler B., Schmidt J., Nawroth P.P., Opelz G. (2008). Metabolic control improves long-term renal allograft and patient survival in type I diabetes. J. Am. Soc. Nephrol..

[19] Weiss A.S., Smits G., Wiseman A.C. (2009). Twelve-month pancreas graft function significantly influences survival following simultaneous pancreas-kidney transplantation. Clin. J. Am. Soc. Nephrol..

[20] Morath C., Zeier M., Dohler B., Schmidt J., Nawroth P.P., Schwenger V., Opelz G. (2010). Transplantation of the type I diabetic patient: The long-term benefit of a functioning pancreas allograft. Clin. J. Am. Soc. Nephrol..

[21] Bunnapradist S., Cho Y.W., Cecka J.M., Wilkinson A., Danovitch G.M. (2003). Kidney allograft and patient survival in type I diabetic recipients of cadaveric kidney alone *versus* simultaneous pancreas kidney transplants: A multivariate analysis of the UNOS database. J. Am. Soc. Nephrol..

[22] Waki K., Terasaki P.I. (2006). Kidney graft and patient survival with and without a simultaneous pancreas utilizing contralateral kidneys from the same donor. Diabetes Care.

[23] Mohan P., Safi K., Little D.M., Donohoe J., Conlon P., Walshe J.J., O’Kelly P., Thompson C.J., Hickey D.P. (2003). Improved patient survival in recipients of simultaneous pancreas-kidney transplant compared with kidney transplant alone in patients with type 1 diabetes mellitus and end-stage renal disease. Br. J. Surg..

[24] Rayhil S.C., D’Alessandro A.M., Odorico J.S., Knechtle S.J., Pirsch J.D., Heisey D.M., Kirk A.D., van der Werf W., Sollinger H.W. (2000). Simultaneous pancreas-kidney transplantation and living related donor renal transplantation in patients with diabetes: Is there a difference in survival?. Ann. Surg..

[25] Reddy K.S., Stablein D., Taranto S., Stratta R.J., Johnston T.D., Waid T.H., McKeown J.W., Lucas B.A., Ranjan D. (2003). Long-term survival following simultaneous kidney-pancreas transplantation *versus* kidney transplantation alone in patients with type 1 diabetes mellitus and renal failure. Am. J. Kidney Dis..

[26] Israni A.K., Feldman H.I., Propert K.J., Leonard M., Mange K.C. (2005). Impact of simultaneous kidney-pancreas transplant and timing of transplant on kidney allograft survival. Am. J. Transplant..

[27] Norman S.P., Kommareddi M., Ojo A.O., Luan F.L. (2011). Early pancreas graft failure is associated with inferior late clinical outcomes after simultaneous kidney-pancreas transplantation. Transplantation.

[28] Pascual J., Abramowicz D., Cochat P., Claas F., Dudley C., Harden P., Heeman U., Hourmant M., Maggiore U., Salvadori M. (2014). European renal best practice guideline on the management and evaluation of the kidney donor and recipient. Nefrologia.

[29] Waki K., Sugawara Y., Kokudo N., Kadowaki T. (2012). Long-term pancreas allograft survival in simultaneous pancreas-kidney transplantation by era. Clin. Transpl..

[30] Becker B.N., Brazy P.C., Becker Y.T., Odorico J.S., Pintar T.J., Collins B.H., Pirsch J.D., Leverson G.E., Heisey D.M., Sollinger H.W. (2000). Simultaneous pancreas-kidney transplantation reduces excess mortality in type 1 diabetic patients with end-stage renal disease. Kidney Int..

[31] Reese P.P., Israni A.K. (2009). Best option for transplant candidates with type 1 diabetes and a live kidney donor: A bird in the hand is worth two in the bush. Clin. J. Am. Nephrol..

[32] Kennedy W.R., Navarro X., Goetz F.C., Sutherland D.E.R., Najarian J.S. (1990). Effects of pancreas transplantation on diabetic neuropathy. N. Engl. J. Med..

[33] Fioretto P., Mauer S.M., Bilous R.W., Sutherland D.E.R., Steffes M.W. (1993). Effects of pancreas transplantation on glomerular structure in insulin-dependent diabetic patients with their own kidneys. N. Engl. J. Med..

[34] Robertson R.P. (2010). Islet transplantation a decade later and strategies for filling a half-full glass. Diabetes.

[35] Gaston R.S., Alveranga D.Y., Becker B.N., Distant D.A., Held P.J., Bragg-Gresham J.L., Humar A., Ting A., Wynn J.J., Leichtman A.B. (2003). Kidney and pancreas transplantation. Am. J. Transplant..

[36] Boucek P., Saudek F., Pokorna E., Vitko S., Adamec M., Koznarova R., Lanska V. (2002). Kidney transplantation in type 2 diabetic patients: A comparison with matched non-diabetic subjects. Nephrol. Dial. Transplant..

[37] Cosio F.G., Hickson L.J., Griffin M.D., Stegall M.D., Kudva Y. (2008). Patient survival and cardiovascular risk after kidney transplantation: The challenge of diabetes. Am. J. Transplant..

[38] Sampaio M.S., Kuo H.T., Bunnapradist S. (2011). Outcomes of simultaneous pancreas-kidney transplantation in type 2 diabetic recipients*.*. Clin. J. Am. Soc. Nephrol..

[39] Light J., Tucker M. (2013). Simultaneous pancreas kidney transplants in diabetic patients with end-stage renal disease: The 20-yr experience. Clin. Transplant..

[40] Margreiter C., Resch T., Oberhuber R., Aigner F., Maier H., Sucher R., Schneeberger S., Ulmer H., Bösmüller C., Margreiter R. (2013). Combined pancreas-kidney transplantation for patients with end-stage nephropathy caused by type-2 diabetes mellitus. Transplantation.

[41] Wiseman A.C., Gralla J. (2012). Simultaneous pancreas kidney transplant *versus* other kidney transplant options in patients with type 2 diabetes. Clin. J. Am. Soc. Nephrol..

[42] Mange K.C., Joffe M.M., Feldman H.I. (2001). Effect of the use or non-use of long-term dialysis on subsequent survival of renal transplants from living donors. N. Engl. J. Med..

[43] Kasiske B.L., Snyder J.J., Matas A.J., Ellison M.D., Gill J.S., Kausz A.T. (2002). Preemptive kidney transplantation: The advantage and advantaged. J. Am. Soc. Nephrol..

[44] Becker B.N., Rush S.H., Dykstra D.M., Becker Y.T., Port F.K. (2006). Preemptive transplantation for patients with diabetes-related kidney disease. Arch. Intern. Med..

[45] Pruijm M.T., De Fijter H.J.W., Doxiadis I.J., Vandenbroucke J.P. (2006). Preemptive *versus* non-preemptive simultaneous pancreas-kidney transplantation: A single-center, long-term follow-up study. Transplantation.

[46] Huang E., Wiseman A., Okumura S., Kuo H.T., Bunnapradist S. (2011). Outcomes of preemptive kidney with or without subsequent pancreas transplant compared with preemptive simultaneous pancreas/kidney transplantation. Transplantation.

[47] Browne S., Gill J., Dong J., Rose C., Johnston O., Zhang P., Landsberg D., Gill J.S. (2011). The impact of pancreas transplantation on kidney allograft survival. Am. J. Transplant..

[48] Wiseman A.C., Huang E., Kamgar M., Bunnapradist S. (2013). The impact of pre-transplant dialysis on simultaneous pancreas-kidney *versus* living donor kidney transplant outcomes. Nephrol. Dial. Transplant..

[49] Ishani A., Ibrahim H.N., Gilbertson D., Collins A.J. (2003). The impact of residual renal function on graft and patient survival rates in recipients of preemptive renal transplants. Am. J. Kidney Dis..

[50] Levin N.W., Kotanko P., Eckardt K.U., Kasiske B.L., Chazot C., Cheung A.K., Redon J., Wheeler D.C., Zoccali C., London G.M. (2010). Blood pressure in chronic kidney disease stage 5D-report from a kidney disease: Improving global outcomes controversies conference. Kidney Int..

[51] Duong U., Mehrotra R., Molnar M.Z., Noori N., Kovesdy C.P., Nissenson A.R., Kalantar-Zadeh K. (2011). Glycemic control and survival in peritoneal dialysis patients with diabetes mellitus. Clin. J. Am. Soc. Nephrol..

[52] Holdaas H., Holme I., Schmieder R.E., Jardine A.G., Zannad F., Norby G.E., Fellström B.C., AURORA Study Group (2011). Rosuvastatin in diabetic hemodialysis patients. J. Am. Soc. Nephrol..

[53] Marz W., Genser B., Drechsler C., Krane V., Grammer T.B., Ritz E., Stojakovic T., Scharnagl H., Winkler K., Holme I. (2011). Atorvastatin and low-density lipoprotein cholesterol in type 2 diabetes mellitus patients on hemodialysis. Clin. J. Soc. Nephrol..

[54] Kalantar-Zadeh K., Cano N.J., Budde K., Chazot C., Kovesdy C.P., Mak R.H., Mehrotra R., Raj D.S., Sehgal A.R., Stenvinkel P. (2011). Diets and enteral supplements for improving outcomes in chronic renal disease. Nat. Rev. Nephrol..

